# A Comparison of the Practical Results Growing out of Physiological and Pathological Experiments with Clinical Experience

**Published:** 1884-08

**Authors:** 


### Editorial.

### A COMPARISON OF THE PRACTICAL RESULTS GROWING OUT OF PHYSIOLOGICAL AND PATHOLOGICAL EXPERIMENTS WITH CLINICAL EXPERIENCE.

This subject urges itself upon our attention at present from the tendency to ignore the facts of clinical observation in grasping after the fruits of the laboratory or the records of experimental investigations upon inferior animals. These fields of labor all have their importance, but it should always come home to the inquirer after truth that the human organism differs in many respects from that of other animals; and we are impressed just now with the peculiarities that differentiate the organizations of the separate orders of creation, by the intensification of the rabies virus in rabbits and guinea pigs, while it becomes mitigated in the monkey, as exhibited in the experiments of Pasteur.

The manifestations of physiological and pathological developments in the hog, from the continued use of alcohol, by M. Dujardin Baumetz, differ so widely from the effects upon man, that we must attribute the contrast to peculiar susceptibilities on the part of swine to suffer from daily dosing with small quantities of alcohol mixed with their food. It is well known that various articles which are poisonous to some classes of animals are eaten with impunity by others; and hence, however desirable it might be to prove chronic alcohol poisoning so as to impress mankind with the danger of using intoxicating drinks, it is not a legitimate inference from influencing hogs injuriously, that similar bad effects ensue with man; and unfortunately there is no lack of human subjects to test it.

The results of observations made upon different men or the same man under various conditions, while using alcohol or another agent calculated to modify the functions of digestion and assimilation, differ so much as to convince us of the greatest caution in drawing conclusions from experiments with the inferior orders of creation. The influence of a medicinal article in health cannot be a guide to its effects in disease; and hence it becomes a matter of the greatest importance to note the modifications induced by special modes of treatment during the progress of different disorders of the human organization, which embodies clinical observation.

With all the help of the improved modes of examination at the bed-side so as to diagnosticate diseases, it behooves the practitioner to set aside hypothesis and preconceived theories as to the operation of medicines in the case of disease, and note closely all the steps in the therapeutic appliances which have accumulated rapidly in these latter days of progressive enterprise. It becomes us to overhaul the properties of drugs in their simple elementary form as compared with the new pharmaceutical preparations, which in some instances have sacrificed efficiency to elegance. But above all, it is incumbent upon the medical profession to keep faithful records of the treatment of cases, with the results, whether favorable or otherwise, and report those of importance for mutual benefit. Clinical experience consists in observing closely, recording promptly, reflecting carefully and reporting faithfully upon the treatment of cases.

